# The Role of Myeloid-Derived Suppressor Cells (MDSC) in Cancer Progression

**DOI:** 10.3390/vaccines4040036

**Published:** 2016-11-03

**Authors:** Viktor Umansky, Carolin Blattner, Christoffer Gebhardt, Jochen Utikal

**Affiliations:** 1 Skin Cancer Unit, German Cancer Research Center (DKFZ), Heidelberg 69120, Germany; c.blattner@dkfz.de (C.B.); c.gebhardt@dkfz.de (C.G.); j.utikal@dkfz.de (J.U.); 2 Department of Dermatology, Venereology and Allergology, University Medical Center Mannheim, Ruprecht-Karl University of Heidelberg, Mannheim 68167, Germany

**Keywords:** myeloid-derived suppressor cells, myelopoiesis, tumor microenvironment, immunosuppression, therapeutic targeting

## Abstract

The immunosuppressive tumor microenvironment represents not only one of the key factors stimulating tumor progression but also a strong obstacle for efficient tumor immunotherapy. Immunosuppression was found to be associated with chronic inflammatory mediators including cytokines, chemokines and growth factors produced by cancer and stroma cells. Long-term intensive production of these factors induces the formation of myeloid-derived suppressor cells (MDSCs) representing one of the most important players mediating immunosuppression. Moreover, MDSCs could not only inhibit anti-tumor immune reactions but also directly stimulate tumor growth and metastasis. Therefore, understanding the mechanisms of their generation, expansion, recruitment and activation is required for the development of novel strategies for tumor immunotherapy.

## 1. Introduction

Myeloid-derived suppressor cells (MDSCs) represent a heterogeneous population of immature myeloid cells consisting of precursors for granulocytes, macrophages or dendritic cells (DCs) that are accumulated during chronic inflammation and tumor progression [1,2,3,4]. These cells show a broadly distinct phenotype. In mice, MDSCs express both CD11b and Gr1 markers and consist of two major subsets: polymorphonuclear Ly6G^+^Ly6C^lo^ (PMN) and monocytic Ly6G^−^Ly6C^hi^ (M) cells [1,2,3,5,6]. In humans, the same two subsets can be characterized as Lin^−^HLA-DR^−/lo^CD33^+^ or Lin^−^HLA-DR^−/lo^CD11b^+^CD14^−^CD15^+^CD33^+^ for PMN-MDSCs and CD14^+^HLA-DR^neg/lo^ or Lin^−^HLA-DR^neg/lo^CD11b^+^CD14^+^CD15^−^ for M-MDSCs [1,2,7,8,9]. MDSCs derive from the bone marrow hematopoietic precursor cells through the pathologic modulation of myelopoiesis induced by constantly produced inflammatory mediators [1,2,3,4,7] and exhibit remarkable immunosuppressive and tumorigenic activities [1,2,3,10]. These functions include (i) a deprivation of amino acids arginine and cysteine, which are essential for T cell proliferation and anti-tumor reactivity [1,11,12]; (ii) a production of nitric oxide (NO) and reactive oxygen species (ROS) that causes the nitration of T cell receptors (TCR) and chemokines important for T cell migration or inducing apoptosis of T cells and NK cells [1,2,3,13,14]; (iii) an intensive production of interleukin (IL)-10 and transforming growth factor (TGF)-β1 inhibiting immune effector cell functions [1,2,3,11,15]; (iv) an upregulated expression of programmed death-ligand 1 (PD-L1) [1,2,3,16] which can drastically downregulate an anti-tumor T cell-mediated reactivity via interaction with PD-1 receptor expressed on T cells [17]; (v) a reduction of the TCR ζ-chain expression playing an important role in coupling the TCR-mediated antigen recognition to diverse signal transduction pathways [4,18]; (vi) a secretion of angiogenic factors promoting tumor neovascularization [19,20], and (vii) a production of growth factors, matrix metalloproteinases and cytokines stimulating tumor growth and skewing immune reactions towards Th2 type and activation of regulatory T cells (Tregs) [2,21,22]. Therefore, MDSCs can be considered as major players in tumor-mediated immunosuppression.

In this review, we will summarize current knowledge of the MDSC generation, migration and acquisition of strong immunosuppressive activity in the tumor microenvironment and will discuss possible targets that could be used for the neutralization of these cells.

## 2. MDSCs Generation and Expansion during Tumor Progression

Numerous reports published during the last decade described a strong correlation between the development of chronic inflammatory conditions in the tumor microenvironment and generation and expansion of MDSCs [1,2,3,4,18,23,24]. Furthermore, chronic inflammation has been found to be associated with the initiation and progression of various tumors [25]. Although the onset of some other tumors such as malignant melanoma is not generally associated with apparent inflammation, recent publications highlighted the critical importance of particular cytokines and chemokines for their fast progression [26]. Tumor cells are able to produce a variety of inflammatory mediators including granulocyte-macrophage colony-stimulating factor (GM-CSF), granulocyte colony-stimulating factor (G-CSF), macrophage colony-stimulating factor (M-CSF), stem cell factor (SCF), vascular endothelial growth factor (VEGF), TGF-β, tumor necrosis factor (TNF)-α, IL-1β, IL-6, and IL-10 [1,2,3,4,26,27]. The effect of all these factors is combinatorial and dose-dependent. Furthermore, tumor cells can induce the production of these factors by fibroblasts and immune cells in the tumor stroma [1,28]. Moreover, stromal cells can further stimulate the production of inflammatory mediators by tumor cells thereby creating autocrine and paracrine loops in the tumor progression [29]. Altogether, these inflammatory factors can modulate myeloid cells in the tumor microenvironment, and having them delivered distantly to hematopoietic organs can change normal myelopoiesis and skew the differentiation of myeloid cells in favor of MDSCs [2,3,4,10,23,30].

GM-CSF is considered as a major growth factor driving myelopoiesis [31,32], whereas further differentiation to granulocytes or macrophages is mediated by G-CSF or M-CSF, respectively [31]. These growth factors have been shown to be expressed in tumor lesions [18,23,27,33]. Tumor-derived GM-CSF has been demonstrated to play a major role in the generation of MDSCs both in vivo and in vitro [34,35]. Moreover, it has been reported that the effect of GM-CSF is dose-dependent: its low concentrations in the absence of IL-4 resulted in the generation of MDSCs and immature DCs from bone marrow hematopoietic precursors in vitro, whereas in high concentrations, it induced the development of neutrophils and mature DCs [36]. In addition, GM-CSF in combination with IL-6, IL-1β, prostaglandin (PG) E2, TNF-α or VEGF has been reported to mediate the generation of highly suppressive MDSCs from CD33^+^ peripheral blood mononuclear cells isolated from healthy donors [37]. Importantly, GM-CSF and IL-6 allowed a rapid and efficient generation of MDSCs with a strong tolerogenic activity from precursors present in mouse and human bone marrow [38].

VEGF and TGF-β have also been demonstrated to be involved in the regulation of hematopoiesis [39,40]. Both growth factors are produced in high concentrations by many tumor types and display a strong impact on the MDSC generation and expansion [1,2,3,4,10,18]. It has been demonstrated that VEGF secreted by tumor cells interfered with the proliferation, differentiation and maturation of immature granulocyte-macrophage progenitors, causing an inhibition of DC maturation and activation as well as a development of immunosuppressive tumor-associated macrophages (TAMs) [41,42]. In combination with VEGF, TGF-β prevented DC maturation, polarized myeloid cells towards immunosuppressive cells in the tumor microenvironment and participated in the induction of TAMs [43].

Impairment of normal myelopoiesis could be also induced by the alterations of cytokine production [44]. They are commonly present in the tumor microenvironment and are regulating by IL-1β [45,46]. It has been documented that IL-1β accumulated at the tumor site is involved in the MDSC generation in bone marrow and in their migration towards tumor lesions [47,48]. Moreover, IL-1β was found to induce cyclooxygenase (COX)-2 expression [46,49] that together with PGE2 could not only mediate an accumulation of MDSCs and TAMs and stimulate tumor progression but also prevent the maturation and activation of antigen presenting cells at the tumor site [50,51]. IL-1β was also demonstrated to up-regulate the production of TNF-α by myeloid and/or tumor cells in the tumor microenvironment [52] that significantly activates MDSC immunosuppressive functions [53,54]. In addition, IL-1β was reported to stimulate the IL-10 production by MDSCs and to play a role in the induction of IL-5 and IL-13 [3,11]. The latter cytokines could stimulate type 2 immune reactions and recruit MDSCs to the tumor microenvironment [55,56].

IL-6 is another cytokine that is critically important for MDSC generation and survival [1,3,10,11]. A strong link of this factor with chronic inflammation and cancer development has been demonstrated [57]. Increased IL-6 concentrations were shown to correlate with MDSC frequencies and their suppressive functions in tumor-bearing hosts [27,58]. The IL-6 signaling involves the signal transducer and activator of transcription 3 (STAT3), preventing MDSC differentiation and promoting their proliferation [1,2,10,59,60]. In addition, blocking IL-6 or IL-6R in prostate cancer and methylcholanthrene-induced skin squamous cell carcinoma mouse models resulted in the prominent reduction of MDSCs infiltrating tumors and in the suppression of tumor development [58,61].

Numerous publications have described a significant increase in the frequency of circulating M-MDSCs and PMN-MDSCs in patients with melanoma [7,8,9,62,63,64,65,66] and other tumor entities [7,8,9,67] that strongly correlated with tumor burden. Furthermore, circulating M-MDSCs have been reported to provide a negative impact on survival [62,64,66] and inversely correlate with the presence of functional antigen-specific T cells in patients with advanced melanoma [64]. High frequencies of PMN-MDSCs correlate with poor prognosis in patients with breast or colorectal cancer [68,69]. The MDSC frequency in cancer patients increased during tumor development. However, 3–4 weeks after surgical resection of the tumor, the frequency of these cells decreased. These findings are consistent with the fact that the generation of MDSCs is due to the higher production of inflammatory factors secreted mostly by the tumor [70,71].

## 3. MDSC Recruitment into the Tumor Site

Chemokines are small (8–14 kDa), structurally related chemotactic cytokines that regulate trafficking of various cells (including leukocytes) through interactions with specific seven-transmembrane, G protein-coupled receptors. Fifty endogenous chemokines that bind 20 receptors have been described [72]. Chemokines are considered to be key drivers in the development of inflammatory diseases and cancer [73]. The pattern of chemokines involved in MDSC migration into the tumor microenvironment seems to be dependent on the MDSC subset (monocytic or polymorphonuclear) and on the tumor model. The role of chemokine (C-C motif) ligand (CCL) 2 and its receptors in the attraction of M-MDSCs has been well described. In particular, it has been demonstrated that an accumulation of M-MDSCs in several mouse tumor models occurred via an interaction between CCL2 and its receptors, chemokine (C-C motif) receptor (CCR) 2, 4, and 5 [74,75]. Moreover, melanoma-infiltrating M-MDSCs displayed CCR2-dependent immunosuppressive activities in the presence of GM-CSF [74]. In the transplantable prostate cancer mouse model, it has been recently demonstrated that CCL2-CCR2 interaction plays a pivotal role in the recruitment of bone marrow-derived myeloid cells to the blood and their subsequent migration into the tumor site [76,77].

The production of CCL2 but also chemokine (C-X-C motif) ligand (CXCL) 8 (also known as IL-8), and CXCL12 can be induced by PGE2 resulting in a dramatic MDSC accumulation in the ovarian and gastric cancer microenvironment [78,79]. In contrast, the expression of CXCL12 has been found to reduce MDSC recruitment in breast cancer mouse model [80]. Other investigators reported a dominating role of CCL3, CCL5 and CX3CL1 but not CCL2 in the migration of M-MDSC [81] or an importance of CXCL-1 (also known as KC), CCL5 and CCL7 in the MDSC enrichment in mouse colon and liver carcinoma models [82,83]. Recently, it has been published that CCL5 strongly activated hypoxia-inducible factor (HIF)-1α signaling cascades leading to the upregulation of VEGF expression [84]. Importantly, both HIF-1α and VEGF are considered to play a key role in MDSC generation and functions [16,85,86]. Interestingly, comparing various transplantable tumor mouse models, Sawanobori et al. [87] observed that MDSC migration into the tumor site could be mediated by different chemokines.

Therefore, the migration of different MDSC subsets into the tumor site can be strongly determined by the histology and the spectrum of chemokines produced by particular tumors.

## 4. MDSC Activation

Numerous recent studies clearly demonstrated that after the generation and migration to the tumor site, MDSC significantly upregulated their immunosuppressive functions. This activating signal is provided by inflammatory molecules such as interferon (IFN)-γ, IL-1β, IL-4, IL-13, TNF-α, toll-like receptor (TLR) ligands, PGE2 and is mediated by transcription factors STAT1, STAT6 and nuclear factor (NF)-κB as well as by elevation of cyclooxygenase (COX)-2 activity [1,2,11,18,23,24,53].

Notably, many of these inflammatory mediators (including IFN-γ, IL-1β, IL-6, TNF-α, CCL2, CCL3, CCL4, CCL5, etc.) are known be produced and secreted in the process of acute inflammation, inducing a significant activation of T cell-mediated immune reactions [1,88]. However, a long-term secretion and maintenance of the same mediators during chronic inflammation or tumor progression stimulates MDSC generation, enrichment and activation, leading to the inhibition of T cell functions as a feedback mechanism. In particular, although IFN-γ is known to be released by activated T cells and is considered as one of the major mediators of anti-tumor T cell-dependent immune responses [89], it may also stimulate tumor promotion. Thus, long-term production of IFN-γ under the sustained antigenic T cell stimulation results in the stimulation of NO production by MDSCs that represent an important mechanism of their immunosuppressive activity [1,2,3,18,23,90]. Moreover, it has been recently reported that IFN-γ produced by CD8^+^ T cells strongly upregulated the expression of PD-L1, which could drastically suppress anti-tumor function of PD-1^+^ T cells infiltrating tumor lesions [17,91]. Importantly, a signaling through PD-L1/PD-1 interaction has recently been attributed to one of the major mechanisms of MDSC immunosuppressive function [16]. Interestingly, the upregulation of PD-L1 expression on MDSCs in tumor-bearing hosts may be also strongly stimulated by HIF-1α under hypoxia conditions [86] that were earlier reported to activate other immunosuppressive mechanisms of MDSCs [85].

**Figure 1:**
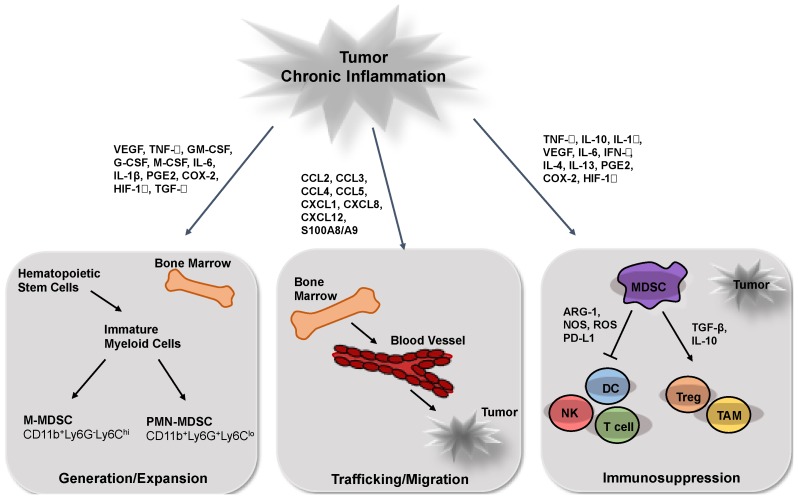
Chronic inflammatory factors stimulate myeloid-derived suppressor cells (MDSC) generation, migration and activation of immunosuppressive functions at the tumor site. Various cytokines and growth factors produced by tumor and stroma cells (such as VEGF, GM-CSF, IL-1β, IL-6, HIF-1α, TGF-β, COX-2, etc.) induce MDSC generation and expansion. Chemokines (like CCL2, CCL3, CCL4, CCL5, CXCL1, CXCL8, etc.) stimulate migration of MDSCs into the tumor microenvironment. At the tumor site, MDSCs undergo activation (via TNF-α, IL-10, IL-1β, IL-6, IFN-γ, COX-2, HIF-1α, etc.) and strongly inhibit anti-tumor reactivity of DC, T and NK cells.

Taken together, inflammatory mediators regulate MDSC expansion, migration and activation in a combinatorial and dose-dependent manner (Figure 1). Moreover, being delivered distantly to various organs in the soluble form or by tumor-derived extracellular vesicles, they can pathologically change myelopoiesis and even convert normal monocytes into highly immunosuppressive MDSCs [1,3,18,23,92].

## 5. MDSCs Stimulate Tumor Progression

**Figure 2:**
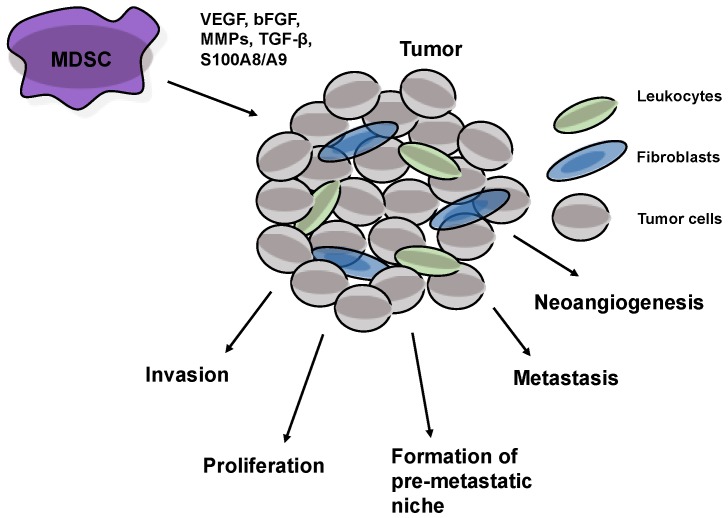
MDSCs support tumor development and metastasis. Soluble factors secreted by MDSCs (such as MMPs, VEGF, TGF-β, etc.) can stimulate tumor neovascularization, invasion, proliferation and metastasis.

There is growing evidence that MDSCs are not only induced, recruited and activated by tumor-derived factors but can also directly support tumor development, neovascularization and metastasis [1,2,3,30,93] (Figure 2). These cells were demonstrated to produce VEGF and basic fibroblast growth factor (bFGF) to promote tumor neoangiogenesis [94,95,96]. MDSCs also participated in tumor neovascularization together with vascular endothelial progenitor cells (EPCs), which are found in different tumor models [95,97]. Moreover, it has been found that MDSCs could even directly incorporate into tumor endothelia, displaying endothelial cell morphology and expressing VEGFR2, a marker for endothelial cells [94].

Furthermore, MDSCs were demonstrated to promote tumor invasion and metastasis by two mechanisms: (i) elevated production of multiple matrix metalloproteinases (MMPs), playing a major role in matrix degradation, and chemokines to create a pre-metastatic environment [95,98,99], and (ii) fusion with tumor cells’ MDSCs promoting the metastatic process [100,101]. Indeed, MDSCs have been shown to infiltrate pre-invasive cancer lesions and to be enriched at the invasive frontier of human cancers [94,102]. In these lesions, MDSCs were able to produce S100A8 and S100A9 induced by VEGF and TGF-β [98]. S100A8/A9 inflammatory proteins have been found not only to attract MDSCs into the tumor microenvironment and enhance their immunosuppressive activity but also to promote the activation of MAPK and NF-κB signaling pathways in tumor cells, stimulating thereby the tumor progression [83,103,104].

## 6. Neutralizing Immunosuppression Induced by MDSCs

A possibility to decrease MDSC numbers and/or immunosuppressive activities leading to the tumor growth delay and the survival prolongation was already demonstrated both in animal models and in cancer patients [6,7,8,9,10,105]. For this purpose, three major strategies were applied: (i) normalization of myelopoiesis; (ii) MDSC depletion or blocking their expansion and activation; and (iii) inhibition of MDSC immunosuppressive functions (Table 1).

Normalization of myelopoiesis includes the prevention of MDSC generation from bone marrow progenitors and the induction of further MDSC differentiation towards mature DCs and macrophages. One of the key targets in preventing MDSC formation is SCF [106,107,132]. The knockdown of SCF with siRNA and inhibition of SCF signaling by anti-c-kit antibodies or with tyrosine kinase inhibitors like sunitinib and sorafenib have been demonstrated to reduce MDSC frequencies in the human bone marrow cells in vitro as well as in murine models of colon and Lewis lung carcinoma that was associated with enhanced anti-tumor reactivity, tumor regression and prolonged survival. In addition, sunitinib has been shown to reverse the MDSC accumulation in patients with renal cell carcinoma (RCC) resulting in the restoration of Th1 cells and a decrease in regulatory T cells [107]. This beneficial effect of sunitinib effect was also detected in the murine RCC model correlated with the suppression of MDSC functions [106]. It has been also reported that the selective pharmacologic inhibition of CSF1R signaling resulted in the decreased tumor angiogenesis associated with reduced recruitment of MDSCs into the tumor site [108]. Moreover, the blockade of CSF1R signaling was found not only to block the MDSC trafficking to tumor lesions but also improve the efficacy of radiotherapy in the prostate cancer model [109]. Furthermore, a recent publication demonstrated that ibrutinib as an irreversible inhibitor of Bruton's tyrosine kinase was able to impair MDSCs’ accumulation in a murine breast cancer model and reduce their immunosuppressive activity reflected by decreased production of NO and expression of indolamine 2,3-dioxygenase [110].

MDSC differentiation into mature myeloid cells could be achieved by the administration of all-trans-retinoic acid (ATRA) [111,112,113] and ultra-low non-cytotoxic doses of chemotherapeutic paclitaxel [114,115]. Although retinoic acid receptors (RAR and RXR) are expressed on various cell types, RARα and RXRα are expressed predominantly on myeloid cells [116]. The combination of ATRA with G-CSF was shown to drive granulocyte differentiation, whereas its combination with Vitamin D stimulated monocyte development [116]. The combination of ATRA with IL-2 administration resulted in a profound decrease in the frequency of circulating MDSCs, in the improvement of DC functions, and tumor-specific T-cell reactivity in patients with metastatic RCC [113]. Another publication reported that ATRA administration into tumor-bearing mice together with human papilloma virus (HPV) therapeutic vaccination decreased MDSC frequencies and functions in the murine HPV-tumor model associated with the activation of tumor-specific T cells and with anti-tumor effects [117]. In addition, the beneficial effect of ATRA applied in combination with DC vaccination has been documented in the clinical trial in the cohort of patients with advanced stage small cell lung cancer [118].

The application of paclitaxel at ultra-low doses to normal mice led to the reduction in the frequency of CD11b^+^Gr1^+^ immature myeloid cells associated with the elevation of NK cell numbers and their ability to produce IFN-γ [119]. Moreover, paclitaxel enhanced the efficiency of peptide vaccination in these mice [119]. In melanoma bearing *ret* transgenic mice, paclitaxel administration induced a significant inhibition of chronic inflammatory factors and MDSC frequencies and functions in melanoma lesion correlated with a partial recovery of tumor-specific T cell responses, leading to profound anti-melanoma effects [115]. Upon the treatment of in vitro generated MDSCs with nanomolar concentrations of paclitaxel, they were demonstrated to differentiate towards DCs in a TLR-4-independent manner [114]. In contrast, paclitaxel failed to induce MDSC apoptosis or affect the MDSC generation from the bone marrow precursor cells.

Direct selective elimination of MDSCs can be achieved by the administration of gemcitabine [120] or 5-fluorouracil [121]. Using several cancer models, it has been found that these chemotherapeutical agents depleted MDSCs without toxic effects on other leukocyte subsets, resulting in markedly enhanced anti-tumor efficacy. The prevention of MDSC trafficking towards tumor lesions is based on the targeting of tumor-derived chemokines. Prostate and breast carcinomas, melanomas, colorectal cancer and Lewis lung carcinoma were found to produce various chemokines (including CCL2, CCL3, CCL4, CCL5, etc.), which were described to attract MDSCs and to maintain their suppressive activity [76,77,78,79,80]. Direct CCL2 targeting [122] or the inhibition of its production [123] has been reported to decrease the frequency of tumor-infiltrating MDSCs, to restrict neoangiogenesis and to suppress the growth of transplantable tumors.

Once migrated into the tumor microenvironment, MDSCs may affect anti-tumor reactivity of T and NK cells by various mechanisms [1,2,3,4,18,23,24]. Among them, the activation of inducible NO synthase (iNOS) and arginase (ARG)-1 plays a key role. Production catalyzed by iNOS was not demonstrated (i) to induce a nitration of T cell receptors in situ [1,2,3,6]; (ii) to target distinct signaling pathways resulting in the inhibition of cytokine production required for T cell functions [14]; (iii) and to mediate T cell apoptosis [14,124]. The activation ARG-1 induced a deprivation of L-arginine, which is not produced by T cells and is critical for protein synthesis [133]. Importantly, the blockade of the activity of phosphodiesterase (PDE)-5 has been reported to increase intracellular concentrations of cyclic guanosine monophosphate (cGMP) resulting in the inhibition of both iNOS and ARG-1 activities [134]. Based on these observations, PDE-5 inhibitors such as sildenafil, tadalafil and vardenafil have been proposed for the inhibition of MDSC immunosuppressive functions [134,125]. The chronic sildenafil administration with the drinking water was reported to cause a significant reduction in the NO production and in the expression of ARG-1 associated with the restoration of tumor-specific CD8 T cell responses and a significantly prolonged survival of tumor-bearing mice [27,125,134]. Moreover, sildenafil could strongly diminish chronic inflammation in the metastatic lymph nodes indicated by a decrease in the production of IL-1β, IL-6, VEGF, GM-CSF, CCL2, CCL3 and S100A9 [27]. In addition, the successful application of tadalafil in a patient with end-stage relapsed/refractory multiple myeloma [126] as well as in clinical trials involving head and neck cancer patients has been recently documented [127,128].

Besides PDE-5 inhibitors, the activity of iNOS and ARG-1 was found to be blocked by corresponding inhibitors [70,125] or by nitroaspirin [129] leading to the stimulation of T cell functions and anti-tumor effects. Interestingly, some agents that prevented MDSC migration towards tumors could also inhibit MDSC immunosuppressive function. In particular, the inhibition of COX-2 activity and PGE2 production has been reported to reduce the CXCR4/CXCL12 and CXCR1-CXCR2/CXCL8-mediated MDSC trafficking [78,79] and to impair the MDSC-mediated immunosuppression by reducing the production of ROS and NO or the expression of ARG-1 in these cells [130].

MDSC numbers and the immunosuppressive pattern could be also modulated by negative checkpoint inhibitors that are widely used for tumor immunotherapy. Thus, in melanoma patients treated with Ipilimumab, decreased amounts and immunosuppressive functionality of both monocytic and polymorphonuclear MDSCs correlated with beneficial therapeutic effects [65,131,135,136,137]. Moreover, non-responding patients showed also elevated serum levels of inflammatory molecules S100A8/A9 and high mobility group box 1 (HMGB1), suggesting that MDSC and chronic inflammatory factors can be not only therapeutic targets in cancer patients but also serve as new biomarkers detecting the group of advanced melanoma patients who may benefit from Ipilimumab therapy [135].

## 7. Conclusions

The role of MDSCs in tumor progression is well-documented. These cells were found to be generated only under pathological conditions such as chronic inflammation and cancer. Established tumors are able to produce multiple factors that impair the myelopoiesis favoring the MDSC formation, trafficking to the tumor site and their activation. Being one of the most potent immunosuppressive cells, MDSCs promote tumor progression by inhibiting the anti-tumor functions of T and NK cells. On the other hand, MDSCs are able to stimulate tumor development directly by promoting neovascularization and tumor cell invasion and by creating a pre-metastatic environment. It is obvious that the efficiency of different immunotherapeutic strategies will be strictly dependent on the neutralization of MDSC-induced immunosuppression. Even adoptively transferred activated tumor-specific CD8 T cells either will develop anergy or even undergo apoptosis, being migrated into an immunosuppressive tumor microenvironment. Therefore, understanding the mechanisms and key regulators of MDSC generation, trafficking and activation is critically important to overcoming immunosuppression and achieving better therapeutic results in cancer patients.

## Figures and Tables

**Table 1:** Therapeutic strategies to inhibit MDSC immunosuppressive activity.

Therapeutic Strategies	References
1. Prevention of MDSC generation and migration	[106,107,108,109,110,111,112,113,114,115,116,117,118,119,120]
2. MDSC depletion or blocking their expansion and activation	[120,121,122,123,124]
3. Inhibition of MDSC immunosuppressive functions	[125,126,127,128,129,130,131]

## Abbreviations

The following abbreviations are used in this manuscript:

ARG	arginase
ATRA	all-trans-retinoic acid
CCL	C-C motif ligand
CCR	C-C motif receptor
cGMP	cyclic guanosine monophosphate
COX	cyclooxygenase
CXCL	C-X-C motif ligand
DCs	dendritic cells
EPCs	endothelial progenitor cells
G-CSF	granulocyte colony-stimulating factor
GM-CSF	granulocyte-macrophage colony-stimulating factor
HIF-1α	hypoxia-inducible factor-1α
HMGB1	high mobility group box 1
HPV	human papilloma virus
IFN	interferon
IL	interleukin
iNOS	inducible NO synthase
M	monocytic
M-CSF	macrophage colony-stimulating factor
M-CSF	macrophage colony-stimulating factor
MDSCs	myeloid-derived suppressor cells
MMPs	matrix metalloproteinases
NO	nitric oxide
PD	programmed death
PDE	phosphodiesterase
PG	prostaglandin
PMN	polymorphonuclear
RCC	renal cell carcinoma
ROS	reactive oxygen species
SCF	stem cell factor
STAT	signal transducer and activator of transcription
TAMs	tumor-associated macrophages
TCR	T cell receptor
TGF	transforming growth factor
TLR	toll-like receptor
TNF	tumor necrosis factor
Treg	regulatory T cells
VEGF	vascular endothelial growth factor
